# Evaluation of the PARACHUTE-HF trial: role of sacubitril/valsartan compared to enalapril on outcomes in patients with heart failure from Chagas’ disease

**DOI:** 10.1007/s10741-026-10617-3

**Published:** 2026-03-30

**Authors:** Dmitry Abramov, Abdul Mannan Khan Minhas, Tamas Alexy, Patricia Oliveira Guimaraes, Mitja Lainscak, Peder L. Myhre

**Affiliations:** 1 Division of Cardiology, Loma Linda University Health, Loma Linda, CA USA; 2 Section of Cardiology, Department of Medicine, Baylor College of Medicine, Houston, TX USA; 3 Department of Medicine, Division of Cardiology, University of Minnesota, Minneapolis, MN USA; 4 Hospital Israelita Albert Einstein, São Paulo, Brazil; 5 Faculty of Medicine, University of Ljubljana, Ljubljana, Slovenia; 6 General Hospital Murska Sobota, Murska Sobota, Slovenia; 7 Department of Cardiology, Akershus University Hospital, Lørenskog, Norway; 8 Institute of Clinical Medicine, K.G. Jebsen Center for Cardiac Biomarkers, University of Oslo, Oslo, Norway; 9 Department of Cardiology, Oslo University Hospital Rikshospitalet, Oslo, Norway; 10 Division of Cardiovascular Medicine, Loma Linda University Medical Center, 2068 Orange Tree Lane, Suite 215, Redlands, 92374 CA USA

**Keywords:** Chagas disease, Chagas cardiomyopathy, Heart failure, Sacubitril/valsartan

## Abstract

Heart failure is a common manifestation of Chagas’ disease and results in significant morbidity and mortality in affected patients. In light of limited data on therapeutic options for heart failure due to Chagas’ disease, the PARACHUTE-HF trial aimed to compare the effects of sacubitril/valsartan versus enalapril on clinical endpoints and changes in natriuretic peptides in this population. This mini-review contextualizes the results of the trial, which demonstrated that sacubitril/valsartan led to significant improvements in outcomes driven primarily by a reduction in natriuretic peptides.

Chagas’ disease, caused by a trypanosomal infection (T. cruzi), is endemic in many Latin American countries with a global prevalence of about 6 million people [1]. While Chagas’ disease can affect numerous organ systems, chronic Chagas’ cardiomyopathy is the most common, affecting 20–30% of patients [1]. The pathophysiology of Chagas’ cardiomyopathy is likely related to chronic myocarditis due to microvascular and perivascular inflammation resulting in dilated left ventricle and impaired ejection fraction. Compared to other etiologies of heart failure with reduced ejection fraction (HFrEF), Chagas’ cardiomyopathy has been associated with higher morbidity and mortality as well as lower quality of life despite patients having lower prevalence of cardiovascular risk factors such as hypertension and diabetes [2]. Although the prevalence of both Chagas’ disease overall and heart failure due to Chagas’ disease have decreased over time with better public health strategies, chronic Chagas’ cardiomyopathy remains a significant public health burden in endemic regions as well as in non-endemic regions due to migration [3–5].

Regarding treatment, anti-protozoal therapy targeting T. Cruzi has been used to reduce the circulating parasite burden, but whether it improves outcomes in patients with Chagas’ cardiomyopathy remains uncertain [6, 7]. Heart failure with reduced ejection fraction caused by Chagas’ disease is therefore generally managed with the same guideline-directed medical therapy (GDMT) as other types of HFrEF [8]. Prior small studies and subgroup analyses of randomized trials evaluating components of GDMT in patients with HFrEF due to Chagas’ disease have demonstrated potential benefits of standard GDMT such as enalapril, carvedilol, and ivabradine. Additionally, a post-hoc analysis of a cohort of 113 patients with HFrEF due to Chagas’ disease from the PARADIGM-HF study suggested a potential benefit of sacubitril/valsartan compared to enalapril (HR 0.63 [95%CI 0.31–1.28] for the primary endpoint of cardiovascular death/HF hospitalization), but the analysis was vastly underpowered and has been interpreted with caution [9]. Recently, a small randomized single-center trial of sacubitril/valsartan versus enalapril in patients with HFrEF due to Chagas’ disease, ANSWER-HF, demonstrated improvements in natriuretic peptides but not clinical endpoints over a 6-month period [10]. Despite these prior small trials and subgroup analyses, no prior large trial has demonstrated clear benefit for a particular component of GDMT on clinical endpoints in this population.

Consequently, the PARACHUTE-HF (Prevention And Reduction of Adverse outcomes in Chagasic Heart failUre Trial Evaluation) trial was designed to prospectively evaluate the effect of sacubitril/valsartan versus enalapril in patients with HFrEF attributed to Chagas’ disease [1, 11]. Key inclusion criteria were clinical heart failure, left ventricular ejection fraction ≤ 40%, New York Heart Association (NYHA) functional class II-IV, and NT-proBNP ≥ 600 pg/mL(BNP ≥ 150 pg/mL), or ≥ 400 pg/mL (BNP ≥ 100 pg/mL) with a hospitalization for HF within the preceding 12 months. Two or more serologic tests were required to confirm the diagnosis of Chagas’ disease. Key exclusion criteria were systolic hypotension (< 95 mmHg), hyperkalemia (> 5.2 mmol/L), advanced hepatic disease, estimated glomerular filtration rate < 30 ml/min/1.73m^2^, and other complications of Chagas’ disease such as severe gastrointestinal manifestations. Patients using sacubitril/valsartan in the past 3 months were excluded, while there were no restrictions related to prior angiotensin converting enzyme inhibitor (ACEi) or angiotensin receptor blocker (ARB) use. Eligible patients were randomized to sacubitril/valsartan or enalapril with a goal to achieve the target dose of sacubitril/valsartan 200 mg twice daily or enalapril 10 mg twice daily. The trial was open-label and did not include a run in-period. The primary endpoint was a hierarchical composite of: time to cardiovascular death, time to first HF hospitalization, or a relative change in natriuretic peptides between baseline and week 12—analyzed using a win ratio. The trial was event-driven and the statistical analysis considered both clinical endpoints and natriuretic peptides in the sample size calculations.

The investigators ultimately enrolled 922 patients from over 80 sites in Brazil, Mexico, Columbia, and Argentina; 462 randomized to sacubitril/valsartan and 460 to enalapril. Overall, the mean age was 64 years and 42% were women. The mean baseline ejection fraction was 30% and the median NT-proBNP was 1730 pg/mL. Most patients were in NYHA class II or III. Concurrent HF medication use prior to enrollment was consistent with clinical practice—over 90% were taking a beta-blocker, approximately 80% were taking an ACEi or an ARB, almost three-quarters were taking a mineralocorticoid receptor antagonist, while only 6% were taking a sodium-glucose co-transporter 2 inhibitor [12]. Less than 5% were on concurrent anti-protozoal therapy. Consistent with known higher arrhythmia risk in patients with Chagas’ cardiomyopathy, almost 40% of the PARACHUTE-HF cohort had a history of ventricular arrhythmia, 31% were on amiodarone therapy, and 46% had either a conventional pacemaker, resynchronization therapy, or implantable cardioverter-defibrillator [12].

The trial was stopped after the occurrence of 324 cardiovascular death/HF hospitalization events as prespecified in the event-driven trial protocol, which included a median follow-up of 25.2 months. Analysis of the primary outcomes revealed that sacubitril/valsartan led to significantly more wins as part of the win ratio, with an overall stratified unmatched win ratio of 1.52 (95% CI 1.28–1.82). The win ratio was predominantly driven by reduction in NT-proBNP; log median change in NT-proBNP was − 30.6% in the sacubitril/valsartan arm and − 5.5% in the enalapril arm from baseline to 12 weeks. Cardiovascular events, including cardiovascular death and HF hospitalization, were numerically less frequent in patients receiving sacubitril/valsartan compared with those receiving enalapril (HR 0.91 [95%CI 0.73–1.13]). Event rates in PARACHUTE-HF were similar to what was estimated in power calculations and were significantly higher than the event rates for all patients in the PARADIGM-HF trial in line with worse clinical outcomes for patients with HFrEF due to Chagas’ disease. In terms of safety endpoints, sacubitril/valsartan was well tolerated, with numerically fewer adverse events leading to study drug discontinuation but more symptomatic hypotension (31.6% vs. 27.4%) compared to enalapril.

While these results represent a significant top-line result in the win-ratio of the primary endpoint, several important additional discussion points warrant consideration. The change in NT-proBNP, which was the main win component of the primary endpoint, was assessed at a single time point (12 weeks) into the study and changes in NT-proBNP at subsequent time points remain unclear. While the majority of NT-proBNP reduction with sacubitril/valsartan appears to occur within the first few weeks [13], the longer-term trajectory up to 25 months is unknown. Additionally, sacubitril/valsartan is known to cause rapid, substantial decreases in NT-proBNP levels—often disproportionate to changes in hemodynamics or left ventricular remodeling. This may be related to increased glycosylation of NT-proBNP, which can reduce detection by most assays, potentially contributing to the observed rapid declines [14]. The reductions in NT-proBNP over the first 12 weeks seen in PARACHUTE-HF were consistent with reductions over a similar time period seen in other randomized trials of sacubitril/valsartan vs. enalapril in patients with systolic HF (Table 1). Regarding the clinical components of the primary outcome, there were only numerical reductions in HF hospitalizations or cardiovascular death. The clinical endpoint findings may raise questions about the benefit of sacubitril/valsartan in patients with HFrEF due to Chagas’ disease, but they should be interpreted with caution due to the limited number of events and lack of trial power for clinical outcomes. The clinical effects of sacubitril/valsartan in Chagas’ disease observed in PARACHUTE-HF mirrored the findings of the smaller ANSWER-HF, which similarly noted that sacubitril/valsartan resulted in reduced natriuretic peptides but did not demonstrate significant differences on clinical endpoints including HF hospitalizations and cardiovascular death. Consequently, while these results may suggest attenuated benefits of sacubitril/valsartan potentially due to differences in underlying pathophysiology of cardiomyopathy in the setting of Chagas’ disease compared to other causes of systolic dysfunction [15], larger studies powered for clinical endpoints are needed to better gauge the possible effects of sacubitril/valsartan on endpoints such as HF hospitalizations or cardiovascular death.

**Table 1:** PARACHUTE-HF and prior randomized trials of Sacubitril/Valsartan vs. Enalapril in heart failure with reduced ejection fraction

Patient population	PARACHUTE HF	PARADIGM-HF	PIONEER-HF	EVALUATE-HF
	Chagas’ Cardiomyopathy	Chronic HFrEF	Acute decompensated HFrEF	Chronic HFrEF
Number of Patients	922	8442	881	464
Mean Age (years)	64	64	61	67
Women (%)	42%	22%	28%	24
Baseline EF	30%	30%	25%	34%
Baseline NYHA II/III	96%	95%	88%	89%
Baseline NT-proBNP (pg/mL)	1730	1608	2746	620
Follow-Up	Median 25 months	Median 27 months	Final: 8 weeks	Final: 12 weeks
Change in NT-proBNP (time)	-31% S/V -6% E (at 12 weeks)	-28% S/V -5% E (at 8–10 weeks)	-47% S/V -25% E (at 4–8 weeks)	-37% S/V -5% E (at 12 weeks)
CV Death/HF Hospitalization, HR (95% CI)	0.91 (0.73–1.13)	0.80 (0.73–0.87)	0.58 (0.39–0.87)	Not Reported

*S/V* sacubitril/valsartan, *E* enalapril, *NYHA* New York heart association, *HR* hazard ratio, *CV* cardiovascular, *HF* heart failure

Other important questions about study design also warrant consideration. While the treatment allocation was randomized, the administration of medication post randomization was open-label, which may have influenced determination of endpoints. Additionally, the statistical analysis for the primary endpoint was based on a win-ratio, which allowed natriuretic peptide reduction to potentially play an oversized role compared to clinical endpoints in driving the overall win hazard ratio.

In conclusion, the PARACHUTE-HF trial provides valuable insights into the effects of sacubitril/valsartan compared to enalapril in the understudied patient population with HFrEF due to Chagas’ disease. The study demonstrates that sacubitril/valsartan was safe and led to meaningful reductions in natriuretic peptides levels at 12 weeks from baseline. Although PARACHUTE-HF was not powered to evaluate clinical outcomes, there were numerically fewer events in the sacubitril/valsartan group compared with the enalapril group. Optimizing GDMT, including sacubitril/valsartan, remains the best evidence-based approach for managing patients with HFrEF due to Chagas’ disease.

## Data Availability

No datasets were generated or analysed during the current study.
